# Psoralen Inactivation of Viruses: A Process for the Safe Manipulation of Viral Antigen and Nucleic Acid

**DOI:** 10.3390/v7112912

**Published:** 2015-11-12

**Authors:** Katherine Schneider, Loni Wronka-Edwards, Melissa Leggett-Embrey, Eric Walker, Peifang Sun, Brian Ondov, Travis H. Wyman, MJ Rosovitz, Sherry S. Bohn, James Burans, Tadeusz Kochel

**Affiliations:** 1 National Biodefense Analysis and Countermeasures Center (NBACC), Fort Detrick, MD 21702, USA; 2 Naval Medical Research Center, Silver Spring, MD 20910, USA; peifang.sun2.ctr@mail.mil; 3 University of Maryland, College Park, MD 20742, USA; sbohn1@umd.edu

**Keywords:** virus, inactivation, biocontainment, BSL-4, BSL-3, psoralen, Trizol LS, IFA, safety, biosafety, PCR, reverse transcription, sequencing, antibody, sterility

## Abstract

High consequence human pathogenic viruses must be handled at biosafety level 2, 3 or 4 and must be rendered non-infectious before they can be utilized for molecular or immunological applications at lower biosafety levels. Here we evaluate psoralen-inactivated Arena-, Bunya-, Corona-, Filo-, Flavi- and Orthomyxoviruses for their suitability as antigen in immunological processes and as template for reverse transcription PCR and sequencing. The method of virus inactivation using a psoralen molecule appears to have broad applicability to RNA viruses and to leave both the particle and RNA of the treated virus intact, while rendering the virus non-infectious.

## 1. Introduction

One of the challenges facing researchers in biocontainment laboratories is that all materials moved from a higher level of biocontainment to a lower biocontainment level (Biosafety Level, BSL) must be proven to be sterile, or undergo a valid and verified method of agent inactivation. This involves inoculating a portion of the material or biological agent containing the sample to be moved to a medium capable of supporting growth of the target organism and evaluating for the presence of agent after sufficient incubation time has passed to allow any viable organisms present to amplify to a detectable level (*i.e.*, colonies on a plate, turbidity in broth). The media and assays used to detect viable organisms will vary depending on the target agents. For human pathogenic viruses, sterility testing is an involved process that begins with inoculation of a portion of the sample of interest into a permissive cell culture line. Depending on the sample, which may contain materials toxic to the cell culture line or require an extended incubation to ensure detectable quantities of virus are present, additional rounds of cell culture amplification may be employed. Once sufficient time has passed to allow amplification of surviving virus, a detection assay such as a plaque assay, immunomicrotitration, or immunofluorescence is performed. The detection of viable agent indicates the process of inactivation was insufficient, and the sample may not be moved to a lower biocontainment area.

Sterility testing takes time (in excess of three weeks, depending on the virus), and researchers have sought inactivation methods that can be validated to support a reduced time before a sample can be moved to a lower biocontainment level. In this sense, inactivation refers to the use of a tested and validated method known to sufficiently and repeatedly render a sample sterile. Materials inactivated using a well-documented and validated inactivation procedure may be moved to a lower biocontainment level, transported, and disposed of without a sterility test. An example of a widely accepted inactivation method is autoclaving; with the process performed correctly and the equipment maintained and operating within normal parameters, and with suitable verification of a full, effective cycle such as biological indicators or machine read-outs, the material subjected to the process is considered sterile. Sterility tests are not performed on every sample that undergoes autoclaving as a method of disposal.

Various formulations of formaldehyde have been widely utilized for the inactivation of virus preparations [1,2,3,4]. However, like autoclaving, this method renders the samples unusable for molecular, genomic and/or immunological methods [5,6,7].

Another widely accepted method of agent inactivation is the use of reagents containing phenol and a chaotropic salt (guanidine isothiocyanate or guanidine thiocyanate, commercially available as TRIzol LS or TriPure reagent, respectively) for the inactivation of viral samples [8,9]. The use of TRIzol LS for the inactivation of viral agents has been a standard accepted method for a number of institutions for decades. However, actual sterility testing data supporting this position are difficult to obtain and complicated to interpret, mainly due to the toxic nature of the TRIzol LS/TriPure reagents to cell culture lines.

TRIzol LS is comprised of phenol, an extremely caustic organic solvent, and guanidine isothiocyanate, a chaotropic salt that denatures macromolecules such as DNA, RNA and proteins. Conventional methods to confirm sterility begin with placing a portion of the inactivated sample on a cell culture layer to provide an opportunity for any surviving virus to infect and grow in sufficient quantity to be detectable by observation of cytopathic effects (CPE) or other detection methods. The toxicity of the TRIzol LS in undiluted, treated viral samples causes almost immediate death of the cell culture layer used for sterility testing. Previous studies have diluted the TRIzol LS-inactivated samples 100- and 1000-fold and still observed tissue culture cell death [9]. While the sterility tests in these studies were negative for the presence of viral agents, it must be noted that the tests could only be performed on treated samples that were diluted many thousand fold and were no longer toxic to the tissue culture cells, so the possibility remains in such scenarios that the presence of small numbers of surviving virus would elude detection.

Attempts have been made to remove TRIzol LS prior to sterility testing. Methods such as dialysis and spin column filtration were investigated, but again the caustic nature of the reagent was problematic. Dialysis material is rated only for very small percentages of phenol (well below the concentration present in TRIzol LS-inactivated samples), and membrane filters were not resistant to phenol. Nevertheless, there is wide acceptance of the TRIzol LS method as a biocontainment laboratory standard for the inactivation of viruses based on historical data, precedent, and the known mechanism of action of the inactivating agent.

Investigation into the properties of psoralens led to the realization that this group of compounds might provide a method for inactivating viral agents while preserving structures needed for antigenic activity and nucleic acids for molecular or genomic analysis. Psoralens are small, photoreactive compounds that freely penetrate phospholipid bilayers and intercalate between nucleic acid pyrimidine residues, causing cross-linking and inactivation via inhibition of replication following exposure to UV-A radiation. Psoralens do not appear to interact with proteins and inactivation of viruses with psoralen may leave immunogenic surface epitopes intact [10,11,12]. The photo-crosslinking property of psoralens has been exploited to inactivate microorganisms in the blood supply [13,14,15] for the treatment of skin disorders [16], to inactivate viral pathogens prior to organ transplantation, and for inactivation of viruses for potential vaccines [17,18,19,20]. Neither exposure to psoralens alone nor exposure to long-wave UV light alone was sufficient to inactivate DNA or RNA viruses, and incubation with psoralens in the absence of UV light did not cause observable cell death [12]. We previously used dengue virus as a representative positive single-stranded RNA (ssRNA(+)) genome virus and demonstrated inactivation of the virus with the psoralen compound 4′-aminomethyl-trioxsalen (AMT) [21,22]. The inactivated dengue virus retained the ability to bind to a panel of five monoclonal antibodies specific for Dengue-2 (DENV-2) envelope protein and elicited a T-cell response in vaccinated mice similar to live, non-inactivated virus [10]. Here we extended the inactivation method to multiple virus families, and examined the suitability of psoralen-inactivated viral RNA for molecular biology applications such as reverse transcription PCR and sequencing. The antigenicity of inactivated virus in the production of antivirus antisera was also examined.

## 2. Materials and Methods

### 2.1. Virus Propagation

Orthomyxoviruses were propagated in MDCK cells; Crimean-Congo Hemorrhagic Fever virus (CCHFV) in HepG2 cells and Lassa virus (LASV) was grown in both RK13 and Vero cells. All other viruses were propagated in Vero cells. Eighty to ninety-five percent confluent cultures were inoculated at a multiplicity of infection (MOI) of 0.001 and propagated in Minimal Essential Medium with 10% fetal bovine serum and 1% antibiotic-antimycotic (product number 15240-062, Life Technologies, Carlsbad, CA, USA) at 37 °C and 5% CO_2_. Six to ten days (depending on the virus) post-inoculation, the culture supernatant was collected, centrifuged at low speed to remove cell debris, and used as virus preparation.

### 2.2. Virus Inactivation by Psoralen

DENV-2 was inactivated according to a previously described method [21]. AMT (product number A4330, CAS number 62442-61-9; Sigma-Aldrich, St. Louis, MO, USA) was added at a final concentration of 10 µg/mL to a DENV-2 stock of 10^6.4^ 50% tissue culture infectious dose (TCID_50_) per milliliter. Five-milliliter aliquots of the mixture were transferred into the wells of 6-well plates. The plates were exposed to UV-A radiation (365 nm; Multiple-Ray Lamp, Ultra Violet Products, Upland, CA, USA) for 0, 2, 5, 10, 20, 30, 40, 50, or 60 min at a measured energy level of 200 µW/cm^2^ (Radiometer, Model PMA2100, UVA+B Detector, Model PMA2107, Solar Light, Glenside, PA, USA). Other viruses inactivated in a similar manner included CCHF IbAr10200 (10^5.1^ TCID_50_/mL), Lassa Josiah (10^5.0^ TCID_50_/mL) (LASV), and Middle East Respiratory Syndrome Coronavirus Jordan (10^6.9^ TCID_50_/mL) (MERS-CoV). A UV-A radiation energy level of 1000 µW/cm^2^ and 20 µg/mL AMT was required to fully inactivate Venezuelan Equine Encephalitis TC83 (10^6.7^ TCID_50_/mL) (VEEV), Junin Candid #1 (10^4.2^ TCID_50_/mL) (JUNV), Rift Valley Fever ZH-501 (10^7.8^ TCID_50_/mL) (RVFV), and Ebola Zaire (10^7.3^ TCID_50_/mL) (EBOV). JUNV and RVFV required 90 min UV-A exposure for full inactivation. EBOV required 120 min UV-A exposure for full inactivation. At each time point, an aliquot was removed from the plates and the titer was determined by TCID_50_. Each aliquot was assayed for viability by virus isolation in Vero cells using indirect immunofluorescent antibody (IFA) assay.

### 2.3. TCID_50_

A TCID_50_ assay was adapted from Lennette [23]. Aliquots from each time point were placed into wells of a 96-well plate containing Vero cells at 95% confluency. The aliquots were serially diluted 1:10 in the plates for a total of 11 dilutions. Plates were incubated at 37 °C at 5% CO_2_ for 10 days. Cell monolayers were observed microscopically for the presence of CPE. The TCID_50_ endpoint dilution was calculated by the Reed and Muench method [24].

### 2.4. Antibody Production

Female New Zealand white rabbits from Charles River Laboratories (Wilmington, MA, USA) received injections of viral antigen on days 0, 30, and 60 followed by exsanguination on day 90. The day 0 injection consisted of 250 µL of antigen emulsified with 250 µL of complete Freund’s adjuvant (Sigma-Aldrich). The remaining injections consisted of 250 µL of antigen emulsified with 250 µL of incomplete Freund’s adjuvant (Sigma-Aldrich). The injected virus antigen was clarified supernatant centrifuged at 2000 RPM at 4°C for 15 min. The minimum pre-inactivation or live titer for injected viral antigen was 1.0 × 10^6^ TCID_50_/mL.

### 2.5. Immunofluorescence Assay (IFA)

Viruses were tested by IFA [25,26]. Aliquots of virus from each inactivation test time point were placed into cell culture flasks containing Vero cell monolayers at 95% confluency. Flasks were incubated at 37 °C at 5% CO_2_ for 10 days and periodically observed for CPE. Cultures were allowed to incubate for the maximum possible time to allow any viable virus to have a chance to replicate to detectable levels. After 10 days, a portion of each culture supernatant was placed into new cell culture flasks and incubated under the same conditions for another 10 days and observed for CPE. The resulting cell monolayers were scraped and applied to microscope slides following the method of Hsiung [26]. Briefly, droplets of cell suspension were applied to slides and allowed to dry at room temperature in the biosafety cabinet (BSC). The sides were fixed for 15 min in acetone to permeabilize the cell membrane and secure the cells to the slide. After acetone fixation, the slides were probed with an anti-virus antibody followed by a fluorescein-labeled goat anti-species antibody (Table 1). Slides were then microscopically screened to detect cells exhibiting fluorescence.

**Table 1:** Immunofluorescence assay (IFA) reagents.

Virus	Primary Antibody	Primary Source	Secondary Antibody	Secondary Source
VEEV	Mouse monoclonal	Millipore ^1^	Goat anti-mouse	KPL ^6^
JUNV	Ascitic fluid	ATCC ^2^	Goat anti-mouse	KPL
RVFV	Rabbit polyclonal	NBACC ^3^	Goat anti-rabbit	KPL
MERS-CoV	Rabbit polyclonal	Sino Biological ^4^	Goat anti-rabbit	KPL
EBOV	Rabbit polyclonal	NBACC	Goat anti-rabbit	KPL
DENV	Mouse monoclonal	GenWay Biotech ^5^	Goat anti-mouse	KPL

^1^ EMD Millipore, Billerica, MA, USA; ^2^ American Type Culture Collection, Manassas, VA, USA; ^3^ National Biodefence Analysis and Countermeasures Center, Fort Detrick, MD, USA; ^4^ Sino Biological, Inc. North Wales, PA, USA; ^5^ Genway Biotech Inc. San Diego, CA, USA; ^6^ Kirkegaard & Perry Laboratories, Inc. Gaithersburg, MD, UAS. VEEV: Venezuelan Equine Encephalitis TC83; JUNV: Junin Candid #1; RVFV: Rift Valley Fever ZH-501; MERS-CoV: Middle East Respiratory Syndrome Coronavirus Jordan; EBOV: Ebola Zaire; DENV: dengue virus.

### 2.6. Flow Cytometry

The amount of DENV internalization by host cells was determined in the Raji B-cell leukemic cell line stably expressing the dendritic cell-specific intercellular adhesion molecule-3-grabbing non-integrin (DC-SIGN) molecule [27]. Briefly, 50 µL/well of live and psoralen-inactivated DENV at a concentration of 1.7 × 10^7^ pfu/mL were added to a V-bottom 96-well plate (Corning Incorporated, NY, USA) containing 1 × 10^5^ per 50 µL of Raji-DC-SIGN cells. After 4 h of incubation in a humidified 37 °C CO_2_ incubator, the cells were washed, fixed, and permeabilized using the BD Cytofix/Cytoperm™ kit (BD Biosciences, San Jose, CA, USA) following the manufacturer’s instructions. The fixed cells were stained with a monoclonal antibody to DENV envelope protein (3H5) or an IgG1 isotype control (BD Biosciences) followed by a FITC (fluorescein isothiocyanate) labeled secondary goat-anti-mouse IgG antibody (NBACC). Cells not treated with virus served as the negative control. After 30 min of staining for each step, the cells were sorted on a FACSCANTO II (BD Biosciences). The percent of 3H5 positive cells represented the number of cells that captured and internalized DENV using the DC-SIGN receptor.

### 2.7. Oligonucleotide Primer and Probe Design and Generation

Sequences for virus genes were obtained from Genbank (http://www.ncbi.nlm.nih.gov/genbank). Primers and probes were designed using Integrated DNA Technologies PrimerQuest primer and probe design tool (http://www.idtdna.com/PrimerQuest/Home/Index). All oligonucleotide primers and probes used in this study are listed in Table 2. Oligonucleotide primers and probes were synthesized by Integrated DNA Technologies (Coralville, IA, USA).

**Table 2:** Primers and probes used in this study.

Name	Target	Sequence	Product Size (bp)	Reference
DENV-8 F	NS3	GCTGAAATGGAGGAAGCCCT	637	this study
DENV-8 R		CCCGCTCTTCACCATCTGTT		
DENV-8 P		CAGAGCTGAGCACACCGGGC		
DENV-9 F	NS4B	AGTTCCCCTTCTCGCCATTG	961	this study
DENV-9 R		GAGTGTTCGTCCTGCTTCCA		
DENV-9 P		AAAAGAGCAGCGGCGGGCAT		
DENV-3 F	RdRp/NS5	TACAACATGATGGGAAAGCGAGAGAAAAA	265	[28]
DENV-3 R		GTGTCCCAGCCGGCGGTGTCATCAGC		
DENV-3 P		AAGAGACGTGAGCAGGAAGGAAGGGGGAGC		
DENV-10 F	NS5	GGAGGAGCAATGTATGCCGA	735	this study
DENV-10 R		GTCGCGTCTGTGGAAGTACA		
DENV-10 P		GGTCTTTGCGGGAGACGGCC		
DENV-11 F	NS5/UTR	AGAGAAGACCAATGGTGCGG	488	this study
DENV-11 R		CCTTCCAGCGAGACTACAGC		
DENV-11 P		CTACCTGTGAGCCCCGTCCAAG		
EBOV-Z F	NP	TGGAAAAAACATTAAGAGAACACTTGC	79	[29]
EBOV-Z R		AGGAGAGAAACTGACCGGCAT		
EBOV-Z P		CATGCCGGAAGAGGAGACAACTGAAGC		
JUNV F	NP	CAGTTCATCCCTCCCCAGATC	79	[30]
JUNV R		GGTTGACAGACTTATGTCCATGAAGA		
JUNV P		TGTTCAACGAAACACAGTTTTCAAGGTGGG		
LASV F	GPC	TGCTAGTACAGACAGTGCAATGAG	79	[30]
LASV R		TAGTGACATTCTTCCAGGAAGTGC		
LASV P		TGTTCATCACCTCTTC		
MARV F	VP40	GGACCACTGCTGGCCATATC	103	[31]
MARV R		GAGAACATITCGGCAGGAAG		
MARV P		ATCCTAAACAGGCTTGTCTTCTCTGGGAC TT		
RVFV F	L	TGAAAATTCCTGAGACACATGG	89	[32]
RVFV R		ACTTCCTTGCATCATCTGATG		
RVFV P		CAATGTAAGGGGCCTGTGTGGACTTGTG		

### 2.8. RNA Extraction

Total RNA was extracted from psoralen-treated cell supernatants using the QIAamp Viral RNA Mini Kit (Qiagen, Valencia, CA, USA) according to the manufacturer’s instructions. All extracted RNA samples were eluted in a volume of 120 µL. RNA purity was assessed using a Qubit^®^ 2.0 Fluorometer (Life Technologies) and the Qubit^®^ RNA HS Assay Kit (Life Technologies) according to manufacturer’s instructions. RNA was stored at −80 °C.

### 2.9. Reverse Transcription Real-Time PCR Assay

Reverse Transcription Real-Time PCR assays were performed using the SuperScript^®^ III Platinum One-Step qRT-PCR Kit w/ROX (Life Technologies) according to manufacturer’s instructions, using a final concentration of 0.2 µM/reaction for each of the primers, and 0.1 µM/reaction for each probe. Five microliters of sample were assayed per 50 µL reaction. Each reaction consisted of 1 cycle of 50 °C for 15 min for reverse transcription, followed by 1 cycle of 95 °C for 2 min for reverse transcriptase inactivation, then 45 cycles of 95 °C for 15 s and 60 °C for 75 s. Reverse transcription real-time PCR assays for each target included no-template controls and test samples. All reverse transcription real-time PCR reactions were run on an Applied Biosystems 7900HT or 7500 Fast (Life Technologies) in standard mode.

### 2.10. Sanger Sequencing

PCR amplicons were sequenced using the Applied Biosystems BigDye^®^ Terminator v3.1 Cycle Sequencing Kit and POP-7™ Polymer (Life Technologies) following the manufacturer’s instructions. The reactions were processed on an Applied Biosystems 3130 Genetic Analyzer (Life Technologies). Raw sequences were analyzed using Sequencher 5.3 software (Genecodes, Ann Arbor, MI, USA).

### 2.11. Next Generation Sequencing

Total RNA prepared from psoralen-inactivated DENV was reverse transcribed using random primers and Superscript^®^ III Reverse Transcriptase (Life Technologies) following the manufacturer’s instructions. cDNA was amplified with the Ready-To-Go GenomiPhi V3 Kit (GE Healthcare, Piscataway, NJ, USA), and a Nextera^®^ XT library (Illumina, San Diego, CA, USA) was prepared for sequencing on the Illumina HiSeq platform following the manufacturer’s instructions in protocol “Paired-end 2 × 150 bp” (Illumina), which uses joined fragments of known length to provide more information in regions of repetitive sequence [33,34].

## 3. Results

### 3.1. Inactivation of DENV Using a Psoralen and UV-A

Dengue virus (a representative ssRNA(+) virus) was previously inactivated by 30 min of UV-A exposure in the presence of a psoralen compound, AMT [21,22]. Treated viruses were subjected to plaque assay to demonstrate inactivation and were successfully used as antigen for mouse antibody production. Here, inactivation of DENV was assessed by TCID_50_, repeat TCID_50_ after subculture, and IFA. Again, thirty minutes of exposure to UV-A light in the presence of AMT was sufficient to reduce viability to a level undetectable by TCID_50_ after 10 days of culture. However, sequentially passaged subcultures displayed visible CPE after a second 10 days of culture and virus was detectable by IFA. This is a significant change from the earlier plaque assay data and illustrates that rigorous testing methods must be used to confirm inactivation. Forty minutes of exposure to UV-A light in the presence of AMT was required to fully inactivate DENV as determined by CPE and IFA after two ten-day sequential cultures (Table 3). Viral cultures that were exposed to UV light but not AMT showed no reduction in viability.

**Table 3:** DENV viability assessed by TCID_50_ (50% tissue culture infectious dose), cytopathic effects (CPE) and IFA after inactivation with 4′-aminomethyl-trioxsalen (AMT) plus ultraviolet light.

Minutes UV-A Exposure	UV-A µW/cm^2^	Passage 1			Passage 2	
		Log_10_ TCID_50_/mL	CPE	IFA	CPE	IFA
0	0	6.4	yes	yes	yes	yes
2	400	5.8	yes	yes	yes	yes
5	1000	4.9	yes	yes	yes	yes
10	2000	3.5	yes	yes	yes	yes
20	4000	1.9	yes	yes	yes	yes
30	6000	0	no	no	yes	yes
40	8000	0	no	no	no	no
50	10,000	0	no	no	no	no
60	12,000	0	no	no	no	no

### 3.2. Inactivation of Multiple Additional Viruses Using a Psoralen and UV-A

**Figure 1:**
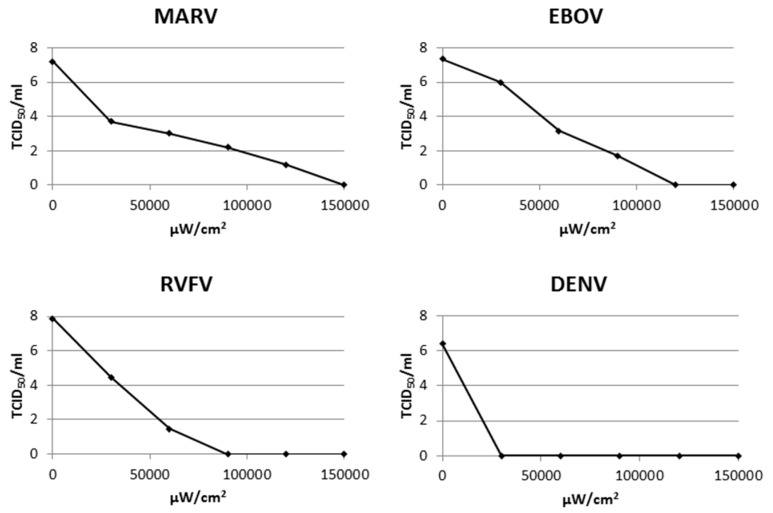
Dose-dependent inactivation of multiple viruses by UV-A in the presence of 4′-aminomethyl-trioxsalen (AMT).

To assess the general applicability of psoralen-mediated virus inactivation, representative viruses from seven families were exposed to UV-A in the presence of AMT (Table 4). Viruses tested included both enveloped and non-enveloped viruses, spherical and filamentous viruses, and both positive and negative sense RNA genomes. Inactivation was achieved for all viruses tested. The rate of titer reduction varied for each virus, suggesting that inactivation was dose-dependent. For example, MARV and EBOV required 100- to 1000-fold more exposure to UV-A in the presence of AMT for inactivation than RVFV and DENV (Figure 1). Each virus treatment was replicated a minimum of eight times.

**Table 4:** Total microwatts UV-A required to inactivate different viruses in the presence of 4′-aminomethyl-trioxsalen (AMT).

Family	Characteristics	Virus	UV-A + AMT Required for Inactivation (µW/cm^2^)
*Alphaviridae*	enveloped, spherical, ssRNA(+)	VEEV	20,000
*Arenaviridae*	enveloped, spherical, ssRNA(-)	LASV	30,000
		JUNV	90,000
*Bunyaviridae*	enveloped, spherical, ssRNA(-)	RVFV	90,000
		CCHFV	4000
*Coronaviridae*	enveloped, spherical, ssRNA(+)	MERS-CoV	6000
*Filoviridae*	filamentous, ssRNA(-)	MARV	150,000
		EBOV	120,000
*Flaviviridae*	enveloped, spherical, ssRNA(+)	DENV	8000
		WNV	2000
		SLEV	1000
		YFV	2000
*Orthomyxoviridae*	enveloped, usually spherical but can be filamentous, ssRNA(-)	H1N1p	1000
		H1N1	1000
		H3N2	2000
		Flu-B	2000

WNV: West Nile; SLEV: St. Louis Encephalitis; YFV: Yellow Fever; H1N1: Influenza A(H1N1)pdm09; H3N2: Influenza A (H3N2); Flu-B: Influenza B.

### 3.3. Viral RNA Retains Molecular Integrity after AMT Treatment

To determine whether the viral RNA isolated from AMT-inactivated viruses was useful for molecular analyses, five representative viruses were tested for competence in reverse-transcription real-time PCR after inactivation with AMT. Junin virus and Lassa virus (*Arenaviridae*), Rift Valley fever virus (*Bunyaviridae*), and Marburg and Ebola viruses (*Filoviridae*) were each successfully amplified using well-characterized and previously published assays (see the materials and methods section). Five microliters of fully inactivated JUNV produced a cycle threshold (Ct) of 32.13, LASV 19.48, RVFV 32.07, MARV 18.08, and EBOV 21.03. Each inactivated virus produced an amplicon of the expected size. Amplicons from LASV, JUNV, RVFV, and EBOV were successfully sequenced and produced identical sequence to untreated virus.

In order to more fully investigate the properties of RNA from an AMT-inactivated virus, five reverse-transcription real-time PCR assays were designed that targeted DENV, a virus with a positive-sense RNA genome. Targets ranging from 119 base pairs (bp) to 961 bp in length were chosen to represent multiple genes and the 3′ untranslated region of the genome. All five targets were successfully amplified from AMT-inactivated DENV. Any photoadducts present in the genome after AMT treatment were not inhibitory to reverse transcription. The cycle in which the fluorescent signal was detectable above background (Ct) of reactions using inactivated virus was similar to the Ct of reactions using live, untreated virus (Table 5). Each DENV amplicon was successfully sequenced and produced sequence identical to that produced by DENV not treated with AMT (data not shown).

**Table 5:** Five PCR targets in DENV and the cycle thresholds (Cts) produced after 0 min (negative control), 30 min, and 60 min exposure to UV-A in the presence of 4′-aminomethyl-trioxsalen (AMT).

Primers	Target	Size (base pairs)	Nucleotide Location	Average Ct/Minutes AMT + UV-A Exposure		
				0	30	60
DENV-8	NS3	637	5206	15.39	17.53	19.30
DENV-9	NS4B	961	7095	17.32	20.22	20.21
DENV-3	RdRp/NS5	120	8923	28.63	29.38	30.31
DENV-10	NS5	735	9148	21.10	22.70	21.35
DENV-11	NS5/UTR	489	10093	17.01	17.25	18.77

**Figure 2:**
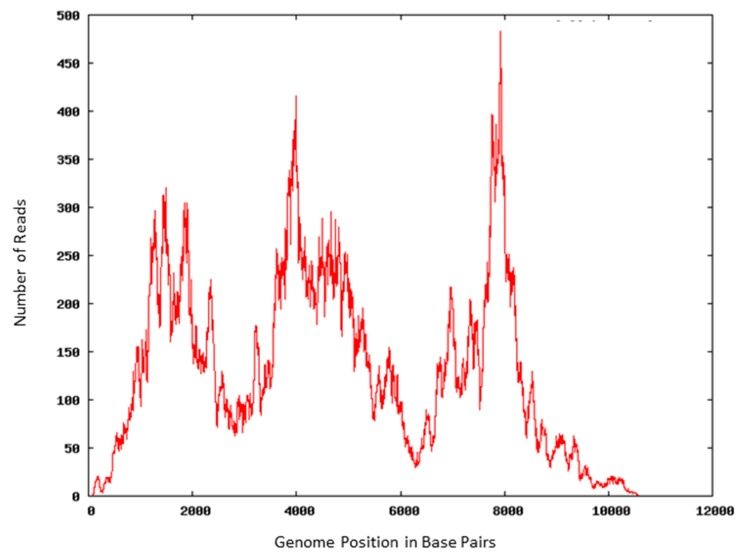
Coverage plot for the Dengue-2 virus genome from the HiSeq reads that mapped to the genome. The differences in coverage between different areas of the genome are probably related to RNA folding, bias in library preparation, or GC content [35], but we cannot rule out the hypothesis that the areas of reduced coverage are due to photoadduct formation after 4′-aminomethyl-trioxsalen (AMT)+UV-A inactivation.

DENV RNA isolated from psoralen-inactivated preparations was reverse-transcribed, amplified, and subjected to whole genome sequencing on the Illumina HiSeq using a paired-end strategy of two 150 bp reads. Approximately 98% of the DENV genome was covered in 13,909 of 203,040,312 total reads. Sequencing coverage varied with the majority of the genome falling between 25- and 400-fold (Figure 2).

### 3.4. Viral Surface Epitopes Maintain Integrity after Psoralen Treatment

AMT-inactivated DENV was previously shown to bind to a panel of monoclonal antibodies specific for DENV-2 envelope protein and to be highly immunogenic in mice and monkeys [10,20]. In this study, additional AMT-inactivated viruses were tested for immunogenicity and antibody generation. Rabbits were inoculated with live or psoralen-inactivated LASV and CCHFV. The collected serum was used as the primary antibody in an IFA against live-virus infected tissue cultures. AMT-inactivated LASV and CCHFV elicited antibodies that specifically recognized untreated LASV and CCHFV, respectively. The antibodies generated against AMT-inactivated viruses were used at the same working dilutions in the IFA as antibodies against untreated virus (Table 6), indicating that titer of each was similar in the serum. This suggests that the antigens on the surface of both live and inactivated viruses were recognized equally well by the rabbit immune system.

**Table 6:** Antibodies produced in response to live and 4′-aminomethyl-trioxsalen (AMT) + UV-A inactivated Lassa virus and Crimean-Congo hemorrhagic fever virus recognized untreated viruses of the same type equally well.

Agent	Rabbit Number	Titer
AMT + UV-A LASV	479876	1:320
AMT + UV-A LASV	479877	1:320
AMT + UV-A LASV	479878	1:320
LASV	479879	1:320
LASV	479880	1:320
LASV	479881	1:640
AMT + UV-A CCHFV	479882	1:160
AMT + UV-A CCHFV	479883	1:320
AMT + UV-A CCHFV	479884	1:640
CCHFV	479885	1:320
CCHFV	479886	1:320
CCHFV	479887	1:320

### 3.5. AMT + UV-A Treated Virus Loses Infectivity While Retaining Receptor Binding

**Figure 3:**
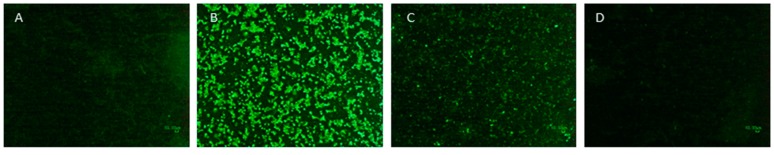
Immunofluorescence assay (IFA) images of Vero cells inoculated with Ebola Zaire virus (EBOV). Anti-EBOV antibody bound virus particles and was in turn bound by fluorescein-labeled goat-anti rabbit. Each image shows fluorescence detected after 10 days in culture: (**A**) negative control, cells only; (**B**) positive control, cells 10 days after inoculation with virus that was exposed to UV-A light but not treated with 4′-aminomethyl-trioxsalen (AMT); (**C**) cells 10 days after inoculation with inactivated virus, fluorescent foci indicate internalized virus particles; and (**D**) cells after inoculation with inactivated virus and subculture four times, for a total of 40 days incubation.

IFA is frequently used to detect viral infection of cultured cells, and has been used in this study to demonstrate that viruses treated with psoralen were fully inactivated. After inactivation with AMT and UV-A, EBOV preparations in cell culture were incubated for 10 days at 37 °C before visualization of viruses using IFA. Some fluorescence was visible within the cells, indicating that the virus had been recognized by cell-surface receptors and internalized. The level of fluorescence was significantly lower than the level observed in the positive control cultures containing live virus (Figure 3), even though similar amounts of virus were inoculated to the flask. This suggests that the amount of inactivated virus present did not increase during the 10-day incubation. After subculture and a second 10-day incubation, the level of fluorescence was very low and nearly indistinguishable from the negative control cultures. After two additional rounds of subculture and incubation for a total of 40 days in culture, there was no discernable virus in the culture inoculated with AMT-treated EBOV (as indicated by lack of CPE and lack of fluorescence by IFA, Figure 3d), indicating that no viral replication had taken place and the virus was completely inactivated by the AMT and UV-A treatment.

### 3.6. AMT-Inactivated Viruses Specifically Bind Cell Receptors

**Figure 4:**
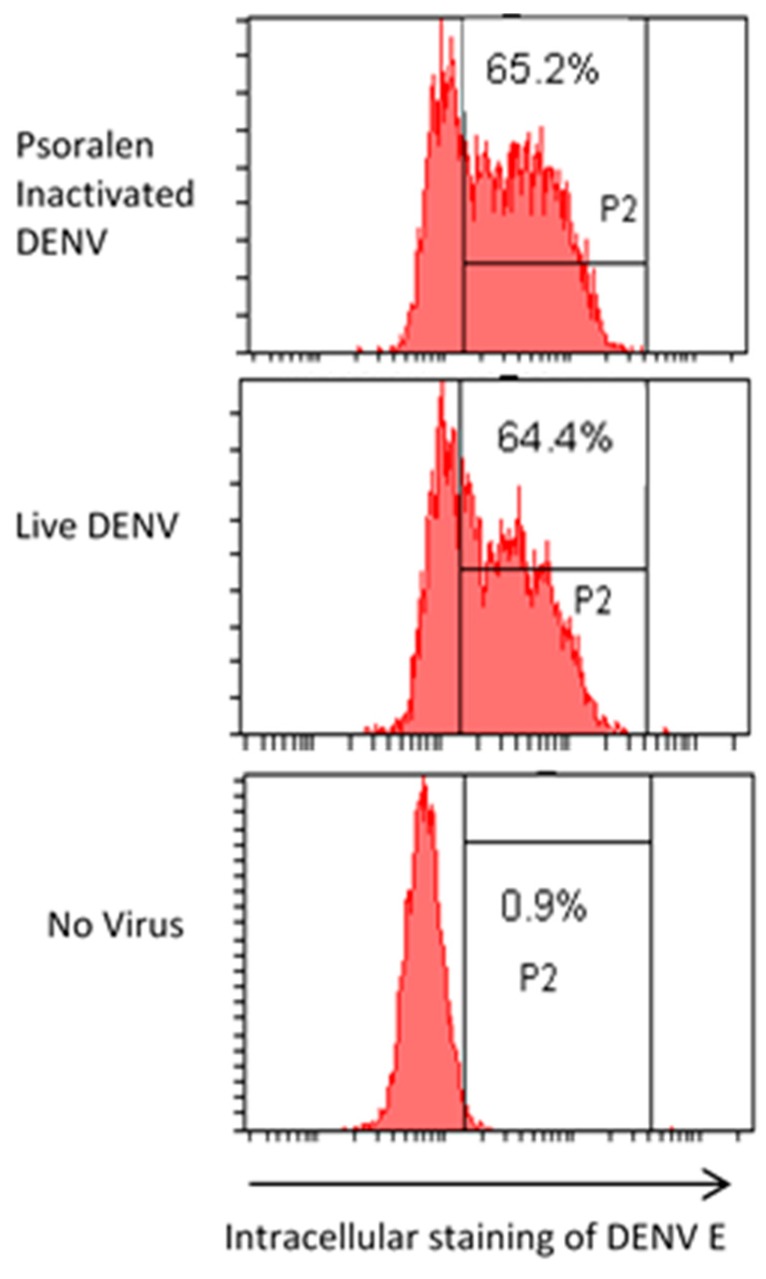
Intracellular staining of dendritic cell-specific intercellular adhesion molecule-3-grabbing non-integrin (DC-SIGN) receptor-expressing cells incubated with inactivated, live, or no Dengue-2 virus (DENV). The proportion of cells to the right of the first vertical line is the proportion that were stained for the DENV E protein (3H5).

Dengue virus enters the cell via specific interaction with clathrin-coated pits [36] and the DC-SIGN receptor [37]. Flow cytometry was used to establish that viruses inactivated with AMT were still competent to interact with DC-SIGN cellular receptors and become internalized. A DC-SIGN expressing cell line [27] was incubated with live and AMT-inactivated DENV. FACS was used to identify cells with internalized virus. A similar proportion of cells showed intracellular fluorescence when incubated with AMT-inactivated virus as when incubated with live DENV (Figure 4), suggesting that the psoralen-inactivated DENV was captured and internalized to the same extent as live DENV in host cells.

## 4. Discussion

The methods of virus inactivation widely in use rely on intense heat or toxic chemicals to render the virus non-infectious. The treatments also denature or crosslink proteins and nucleic acids, preventing inactivated virus preparations from being fully effective as antigens and diminishing or preventing amplification of the nucleic acids for PCR or sequencing. DENV had previously been shown to be inactivated by exposure to UV-A light in the presence of AMT [10]. In this study, this method of inactivating virus preparations was extended to multiple virus families.

The method was effective at inactivating Alpha-, Arena-, Bunya-, Corona-, Filo-, Flavi- and Orthomyxoviruses. Among the families represented were spherical and flexuous viruses, as well as those with positive- and negative-sense RNA genomes, which suggests that the inactivation method will be successful for all RNA viruses. Each virus tested required a different amount of UV-A exposure in the presence of AMT for inactivation, and an increasing proportion of the virus particles became inactivated with increasing exposure to UV-A, indicating a dose-dependent response to the treatment. Rod-shaped viruses that use a polyprotein strategy required orders of magnitude more microwatts of UV-A than most segmented viruses for full inactivation. It is not known why such a large range of susceptibility to AMT + UV-A inactivation was observed. There does not seem to be a consistent set of virus characteristics that leads to the requirement for more exposure to AMT + UV-A for full inactivation. For example some enveloped, spherical viruses tested required only low levels of UV-A in the presence of AMT for full inactivation (CCHFV, H1N1p), while others required quite high levels (JUNV, RVFV). Virus concentration in the inactivation reaction, the type of virus, and the light conditions or UV-A intensity are all parameters that could conceivably alter the inactivation process. It is likely that each individual investigator will have to perform a titration experiment to define the appropriate conditions that consistently produce inactivation in their chosen virus system.

The method of virus inactivation via UV-A exposure in the presence of AMT has multiple benefits. We were able to use AMT-inactivated viruses as targets for antibody detection in IFA and FACS and as antigen for production of new polyclonal antibodies. The polyclonal antibodies produced against inactivated virus worked equally well for detecting untreated virus compared to antibodies produced against live virus preparations. These data taken together suggest that the surface proteins of the inactivated viruses are intact and correctly oriented, allowing full interactions with cells and protein complexes. This will allow viruses inactivated by this method to be used in vaccine development, in antibody generation for research, as positive controls for antibody-based detection assays, or studied by microscopy. The nucleic acids extracted from AMT-inactivated viruses were non-infectious and were sufficiently undamaged in EBOV, JUNV, LASV, MARV and RVFV to achieve successful detection by reverse-transcription real time PCR. The viral nucleic acids were non-infectious and sufficiently undamaged in DENV to be appropriate substrates for real time PCR and both Sanger and next-generation sequencing, thus supporting these methods at lower levels of biocontainment. Library-preparation attempts for next-generation sequencing using standard methods for AMT-inactivated LASV and VEEV were unsuccessful, which may indicate that the crosslinking of these viruses in our hands was too great to allow next-generation sequencing. However, amplicons of up to 971 base pairs were successfully detected by PCR, suggesting that number of photoadducts per genome is less than 1 per 1000 bases, at least in the case of DENV. The 3′ end of the DENV genome was amplified as readily as an internal target, which suggests that the photoadducts are not concentrated near the ends of the virus. Most importantly, the method provides inactive, non-infectious virus preparations that may be reliably sterility tested without attempting to remove toxic chemicals from the preparations.

If this method of viral inactivation is to become a generally accepted method of inactivation, it is necessary to perform multiple site-specific and agent-specific validation studies rigorously testing the method under multiple scenarios to ensure that preparations inactivated by this method are truly non-infectious. The UV-A exposure time and AMT concentration required to inactivate each virus is affected by viral titer and is species-specific, and may even be strain-specific. More data are needed to determine if all members of families of viruses react the same to the treatment. Investigators should work closely with their Health and Safety Offices to plan studies with multiple methods of analysis and a statistically significant number of replicates. In time, this method could provide rapid access to virus preparations and genomic material from pathogenic samples quickly and safely.
